# The impact of early diagnosis of endometriosis on quality of life

**DOI:** 10.1007/s00404-025-07999-4

**Published:** 2025-04-06

**Authors:** Mania Kaveh, Maryam Nakhaee Moghadam, Mojtaba Safari, Shahla Chaichian, Abolfazl Mehdizadeh Kashi, Mehdi Afshari, Kambiz Sadegi

**Affiliations:** 1 Department of Obstetrics and Gynecology, School of Medicine, Amir Al Momenin Hospital, Zabol University of Medical Sciences, Zabol, Iran; 2 Iranian Scientific Society of Minimally Invasive Gynecology, Tehran, Iran; 3 School of Medicine, Zabol University of Medical Sciences, Zabol, Iran; 4 Endometriosis Research Center, Iran University of Medical Sciences, Tehran, Iran; 5 Department of Obstetrics and Gynecology, School of Medicine, Endometriosis Research Center, Hazrat-e Rasool General Hospital, Iran University of Medical Sciences, Tehran, Iran; 6 Department of Community Medicine, School of Medicine, Pediatric Gastroenterology and Hepatology Research Center, Zabol University of Medical Sciences, Zabol, Iran; 7 Department of Anesthesiology, School of Medicine, Amir Al Momenin Hospital, Zabol University of Medical Sciences, Zabol, Iran

**Keywords:** Diagnosis, Endometriosis, Quality of life, Woman, Public health

## Abstract

**Background:**

Endometriosis is one of the most common chronic diseases in women, with a prevalence of up to 10%. The disease particularly affects women of reproductive age. Endometriosis has a significant impact on the patient's quality of life (QoL). In the current study, we aimed to evaluate the role of early diagnosis of endometriosis on patients’ QoL.

**Methods:**

In this longitudinal prospective study, 205 women with endometriosis who were referred to the gynecology department of Amir al-Mominin Hospital (Zabol-Iran) in 2021 were evaluated. Patients were divided into two groups based on the time of diagnosis, including early diagnosis and late diagnosis. An Endometriosis Health Profile (EHP) questionnaire was used to collect information about QoL before and 18 months after treatment. Data were analyzed using SPSSv.26 software and significance level was considered less than 0.05.

**Results:**

In both groups with early and late diagnosis, the QoL scores improved without significant difference (*p* = 0.303). There was a significant difference between lower stages (1 and 2) and higher stages (3 and 4) in terms of treatment effects on patients’ QoL, and higher stages of endometriosis affected patients’ QoL before and after treatment more than lower stages (*P* values < 0.05).

**Conclusion:**

Early or late diagnosis of endometriosis doesn’t affect patients’ QoL and patients benefit from treatment regardless of the time of diagnosis.

## What does this study add to the clinical work

**Table:** 

This study demonstrates that while the timing of endometriosis diagnosis (early vs. late) does not significantly impact quality of life (QoL), treatment effectively improves QoL regardless of diagnosis timing.	
Timely treatment remains the key to enhancing QoL and minimizing the long-term burden of endo.	
Patients with advanced-stage (stages 3 and 4) endometriosis experienced a greater impact on QoL compared to those with earlier stages (1 and 2).	
Treatment led to notable QoL improvements, but the severity of the disease played a crucial.	

## Introduction

Endometriosis is a chronic and complex disease that is recognized as one of the most prevalent gynecological conditions worldwide. It is associated with infertility and chronic pelvic pain, particularly during menstruation and intercourse. The impact of endometriosis on the quality of life of affected individuals and their families is multifaceted [1, 2].

Endometriosis, a chronic condition characterized by the presence of endometrial tissue outside the uterus, is associated with infertility in women, potentially impacting their marital and psychological well-being. The discomfort associated with chronic pelvic pain can hinder daily activities and even interfere with sexual intimacy.A significant aspect of this condition is its economic and financial impact, which is a crucial consideration. The financial burden of treating and managing endometriosis is comparable to that of other chronic diseases, such as type 2 diabetes, Crohn's disease, and rheumatoid arthritis. This impact extends beyond the individual, affecting not only women but also their families [3–5].

The prevalence of endometriosis among women experiencing pelvic pain and during the reproductive period has been documented to range from 10 to 15%. Moreover, among women grappling with chronic pelvic pain or menstrual discomfort, the prevalence of endometriosis escalates to approximately 70% [6]. Vercellini et al. reported that the mean annual cost per patient for healthcare and loss of productivity for patients with endometriosis was approximately €10,000 [7].

The assessment of quality of life (QoL) is a critical component of treatment selection, as the chosen treatment can significantly impact patients' social, physical, and mental well-being [8]. The primary treatment modality entails surgical intervention and endometriosis resection. While the efficacy of surgery in achieving a cure is limited, it has been demonstrated to alleviate pain and enhance the patient's quality of life [9]. Early diagnosis has been demonstrated to positively impact quality of life and alleviate pain symptoms. In their seminal study, Brawn et al. posited that timely diagnosis of endometriosis may serve to mitigate the risk of chronic pain in affected patients [10]. The extant data regarding the effects of the time of diagnosis (early or late) on a patient's quality of life (QoL) and pain are limited [11]. The present study was conducted with the objective of evaluating the effects of early diagnosis on quality of life (QoL) and pain in patients diagnosed with endometriosis.

## Methods

In this longitudinal prospective study, patients referred to the obstetrics and gynecology clinic at Amir al-Mominin Hospital in Zabol, Iran, in 2023 were assessed. The inclusion criteria for the present study included the following: women with a definitive diagnosis of endometriosis confirmed by clinical methods, ultrasound, or laparoscopy; aged between 15 and 54 years at the time of study entry; experiencing symptoms related to endometriosis, including chronic pelvic pain, dysmenorrhea (painful menstruation), dyspareunia (pain during intercourse), or infertility; and no history of other chronic pelvic diseases such as pel inflammatory disease (PID) or irritable bowel syndrome (IBS) that could have symptoms similar to endometriosis, willingness to participate in the study and complete the written consent form, no history of major pelvic surgeries in the past 6 months that could have affected the study results, ability to complete the quality of life questionnaires (EHP) and pain intensity (VAS) before and after treatment. The exclusion criteria encompass patients who do not have a definitive diagnosis of endometriosis or whose diagnosis is based solely on clinical symptoms and not confirmed by standard methods. Furthermore, patients with other chronic diseases, such as irritable bowel syndrome (IBS), autoimmune diseases, or other underlying conditions that could affect their quality of life, are excluded from participation. Additionally, patients who did not complete their treatment during the study period are excluded, including those who did not undergo surgery or did not take the prescribed medications.

Women with a medical history marked by significant pelvic surgical interventions or the presence of malignant reproductive system diseases are excluded from participation.Individuals who did not complete the assessment forms (EHP and VAS questionnaires) or who withdrew from the study are also excluded.Individuals who became pregnant during the study period are excluded due to the potential impact of pregnancy on the symptoms associated with endometriosis and on quality of life.

The decision to establish a five-year cut-off point was informed by a comprehensive review of extant studies, as well as meticulous statistical and clinical evaluations. This strategic choice enabled the harmonization of the research outcomes with existing studies, facilitating a more nuanced interpretation of the consequences of early or late diagnosis on patients' quality of life.

In this study, patients were divided into two groups based on the duration from the onset of symptoms to diagnosis. The first group, designated “early diagnosis,” included patients with a time interval of ≤ 5 years between the onset of symptoms and diagnosis. The second group, designated “late diagnosis,” included patients with a time interval > 5 years between the onset of symptoms and diagnosis [12]. Prior to the initiation of treatment, patients were evaluated for pain using the visual analog scale (VAS) and for quality of life (QoL) via the European Health Profile (EHP) questionnaire. The EHP-30 questionnaire is composed of two sections: the core questionnaire, which contains 30 items across five domains, and the additional section, which contains six questions related to pain (questions concerning limitations in daily and social activities), six questions related to control (questions concerning feelings of hopelessness and helplessness about symptoms), and six questions related to emotional well-being (questions concerning feelings of sadness and mood changes due to the disease), 4 questions related to social support (questions concerning the inability to express emotions and lack of understanding from others), and 3 questions related to self-image (questions concerning the impact of the disease on the patient's appearance and self-confidence) The EHP-30 questionnaire is completed by all patients. Subsequent to a 18-month treatment period, which included surgical or medical interventions, patients were re-evaluated using the VAS and EHP questionnaires.The patient data, encompassing age, stage of endometriosis, QoL before and after 18 months, and VAS before and 18 months after treatment, were meticulously documented and compared between the two groups. The staging of endometriosis was determined based on a pathological assessment of resected endometriosis, utilizing the staging system established by the American Society for Reproductive Medicine (ASRM) [13].

The Persian version of the EPH questionnaire was subjected to a reliability and validity assessment by Nojomi et al. [14].

### Statistical analysis

The data were recorded and analyzed using SPSSv.21 software, with a significance level of less than 0.05.

## Results

In this longitudinal prospective study, 205 women with endometriosis were assessed. The mean age at the time of diagnosis was 31.4 years, with a standard deviation of 8.175. The minimum and maximum age at the time of diagnosis were 15 and 54 years, respectively.

The analysis revealed that 61% of the patients were diagnosed within five years of the onset of symptoms, indicating an early diagnosis, while 39% were diagnosed more than five years after the onset of symptoms, indicating a late diagnosis.

**Fig. 1:**
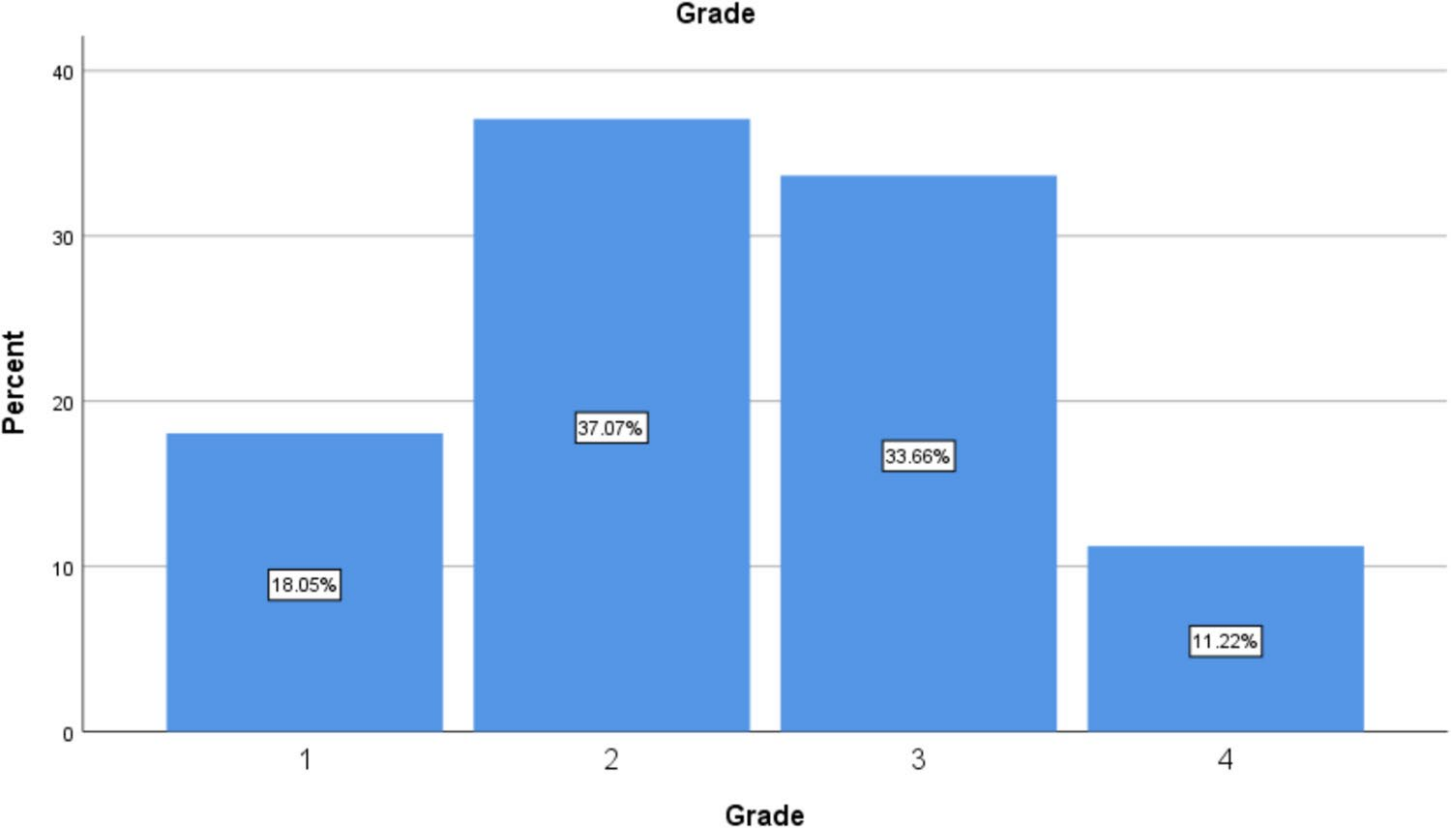
The frequency of endometriosis staging types in the examined patients

With respect to endometriosis staging, 18.05% of patients were diagnosed with stage 1 endometriosis, 37.07% with stage 2, 33.66% with stage 3, and 11.22% with stage 4. Patients with more advanced stages of endometriosis who received early diagnosis and surgical intervention exhibited a greater improvement in their quality of life (QoL) compared to those with less advanced stages (Fig. 1).

The data concerning the quality of life (QoL) before and after treatment, as well as the comparison between the early and late diagnosis groups, are presented in Table 1.

**Table 1:** Results of QoL before and after treatment between the two groups

Time to diagnosis	Mean (standard deviation) of QoL score		Comparison of QoL score before and after treatment (P value) *	Comparison of mean difference of QoL score changes among different diagnosis times (P value)*
	Before treatment	After treatment		
≤ 5 years	51.75 (17.392)	38.92 (17.392)	13.4 (p < 0.0001)	p = 0.303
> 5 years	51.73 (18.523)	41.41 (17.947)	10.3 (p < 0.0001)	
Comparison of quality of life score between two groups P value	0.992	0.234		

*Marginal results by controlling the confounding effect of age, stage, and quality of life score before treatment by ANOVA test

The effects of treatment based on the staging of endometriosis are seen in Table 2.

**Table 2:** Results of the treatment based on staging of endometriosis

Endometriosis stage	Mean (standard deviation) of QoL score		Comparison of QoL score before and after treatment (P value) *	Comparison of mean difference of QoL score changes among different diagnosis times (P value) *
	Before treatment	After treatment		
1&2	49.04 (17.966)	36.60 (18.254)	12.4 (p < 0.0001)	0.114
3&4	55.05 (17.108)	43.22 (16.308)	11.8 (p < 0.0001)	
Comparison of quality of life scores between different stages P value	0.016	0.007		

*Marginal results by controlling the confounding effect of age, stage, and quality of life score before treatment by ANOVA test

## Discussion

In this study that was conducted to evaluate the effects of time of diagnosis endometriosis on a patient’s QoL and pain, we found that the time of diagnosis such as early or late diagnosis did not affect the patient’s QoL or pain. We also assessed the effect of the treatment on the patient’s QoL based on the endometriosis stage and it was found that there was a significant difference between stages 1 and 2 with stages 3 and 4 in terms of the treatment effects on QoL of patients. Based on our findings, higher stages of endometriosis (stages 3 and 4) impact patient’s QoL before and after the treatment more than lower stages (stages 1 and 2).

Early diagnosis of endometriosis is challenging because its clinical manifestations may overlap with other organ involvements like urologic, gastrointestinal, or other gynecologic involvements. On the other hand, patients may consider these manifestations as routine menstrual manifestations and do not refer to a gynecologist [15]. Delay occurs prevalently in about 4–11 years from the onset of symptoms to the definite diagnosis of endometriosis [16]. Soliman et al. mentioned that the mean delay time to diagnosis is 4.4 years [17].

Davenport et al., evaluated causes of delayed diagnosis in patients with endometriosis. They found that considering pathologic symptoms from monthly menstrual symptoms and using self-care, delay to visit gynecologist, lack of oral contraceptive pill use in the diagnostic process, overlap of symptoms with other conditions and variable symptoms, lack of noninvasive diagnostic method were the main causes of delayed diagnosis [18].

Recent studies have emphasized the complex relationship between endometriosis, pain, and mental health. Škegro et al. found that depressive symptoms (44.3%), anxiety (25.3%), and stress symptoms (31.7%) were common in women with endometriosis. They also reported moderate correlations between quality of life scores and depression, stress, and pain levels, highlighting the need for a multidisciplinary treatment approach that considers both physical and psychological aspects of the disease [19].

One of the important points that may be underestimated is the knowledge of physicians, especially general practitioners, about endometriosis. In a study, it was demonstrated that general practitioners did not know enough about endometriosis [20]. In another study, it was found that 74.3% of patients with endometriosis were misdiagnosed with some other differential diagnosis like pelvic inflammatory disease, irritable bowel syndrome, and appendicitis [21]. In the current study, we didn’t assess the causes of delay in the diagnosis of endometriosis but it seems that more policies should be performed to decrease the delay in the diagnosis of endometriosis.

Of the other important point in terms of delayed diagnosis is that a definite diagnosis sometimes needs to be confirmed by pathology and patients do not accept a surgical procedure for this confirmation. This process may also increase the time to diagnosis [22].

Mousa et al. evaluated QoL and diagnostic delay in patients with endometriosis. They mentioned that poorer QoL was associated with a higher rate of diagnosis delay in women with endometriosis. Pain and physical dysfunction decrease the QoL of patients and this process is aggravated by delay in the diagnosis [23]. These findings were different from the findings of the current study. We found that there was no association between delayed diagnosis and QoL and the treatment has a similar impact on patients with early diagnosis and late diagnosis based on QoL. Mousa et al. mentioned that the average delay of diagnosis for all patients was 11.61 years [23]. In the current study, we divided patients into two groups including equal or less than 5 years and more than 5 years that was one of the differences between these two studies.

Furthermore, the role of infertility in worsening mental health outcomes among women with endometriosis has been highlighted. The validation of the EHP-5 questionnaire in Croatia demonstrated significant differences in QoL scores between infertile and fertile women, with infertile women reporting worse outcomes. These findings reinforce the need to address infertility concerns alongside pain management and psychological support [24].

Al Shukri et al. investigated clinical predictors of early diagnosis in patients with endometriosis. They concluded that there was no association between clinical profiles like age at the time of diagnosis and delay in the diagnosis in patients with endometriosis [25]. In the current study, we found that there was no relationship between delayed diagnosis and the patient’s QoL. The findings of these two studies indicate that few parameters have effects on the QoL of patients with endometriosis. Based on our study, only one stage of endometriosis impacts the QoL of patients.

The European Society of Human Reproduction and Embryology (ESHRE) guideline mentioned that there are limited studies about the impact of early versus delayed diagnosis of endometriosis on QoL of patients, but it seems that early diagnosis had better effects on QoL of patients [26]. We found that although early vs. delayed diagnosis had no different impact on the QoL of patients, timely diagnosis and treatment lead to faster relief of patients from pain, and diagnosis of endometriosis at any stage increases the QoL.

Many studies have been conducted regarding the impact of endometriosis and its diagnosis and treatment on the quality of life [27]; but few studies like our study investigated the impact of early diagnosis on QoL and compared it with the results of late diagnosis and it was one of the advantages of the current study.

Several tools have been developed to assess QoL in patients with endometriosis, each with its own strengths and limitations. The Endometriosis Health Profile (EHP) questionnaire, which we utilized in our study, was chosen due to its disease-specific nature, ensuring a more precise evaluation of endometriosis-related symptoms and their impact on QoL. Compared to the Short Form-36 (SF-36), which is a general health questionnaire applicable to various conditions, EHP provides more detailed insights tailored to endometriosis. While SF-36 is widely used for its broad applicability, it may not fully capture the unique challenges faced by endometriosis patients.

Similarly, the World Health Organization Quality of Life (WHOQOL-BREF) questionnaire is another widely used tool that offers a comprehensive evaluation of overall well-being but lacks the disease-specific focus that EHP provides. WHOQOL-BREF is beneficial for comparing QoL across different populations; however, it may not be sensitive to the specific impacts of endometriosis. Given these considerations, the EHP was the most appropriate choice for our study as it allowed us to gather more detailed and relevant data on the QoL of endometriosis patients.

One of the strengths of our study is the comprehensive patient assessment, which involved a thorough evaluation of 205 women with endometriosis. This provided a robust dataset for analysis. The study's longitudinal design allowed us to assess QoL changes over an 18-month period, giving us a clear understanding of long-term effects. Additionally, by comparing different stages of endometriosis, we were able to highlight how the severity of the disease impacts QoL before and after treatment.

However, our study has certain limitations. The limited geographic scope, as the study was conducted in a single hospital in Zabol, Iran, may limit the generalizability of the findings to other regions and populations. We also did not control for other variables that might affect QoL, such as comorbidities or socioeconomic factors. Lastly, the QoL was assessed using self-reported questionnaires, which may introduce bias and variability in responses.

## Conclusion

Our study did not show evidence in favor of early diagnosis and treatment of endometriosis leading to improved QoL, but it can be emphasized that early diagnosis and proper treatment lead to faster relief of patients from pain, and diagnosis of endometriosis at any stage increases the quality of life. Also, early diagnosis decreases complications of endometriosis like infertility, ureter, and bowel involvement.

## Data Availability

No datasets were generated or analysed during the current study.
